# Body position alters kyphosis angle: comparison of supine MRI and prone full-length spine CT scout view in osteoporotic thoracolumbar fractures

**DOI:** 10.3389/fsurg.2026.1740997

**Published:** 2026-04-14

**Authors:** Xiubo Ge, Rui Zhao, Yifei Li, Liang Zhao, Haitao Lu, Haiyang Yu

**Affiliations:** 1 Department of Orthopedics, Fuyang People’s Hospital Affiliated to Anhui Medical University (Fuyang People’s Hospital), Fuyang, Anhui, China; 2 Clinical Research Center for Spinal Deformity of Anhui Province, Fuyang, Anhui, China

**Keywords:** kyphosis, kyphosis flexibility, osteoporotic thoracolumbar fracture, position, thoracolumbar

## Abstract

**Objective:**

To compare the degree of kyphosis among patients with old thoracolumbar fracture kyphosis (OTFK) in various positions and to assess kyphosis flexibility.

**Methods:**

A total of 32 patients with OTFK who met the inclusion criteria were retrospectively included between February 2017 and August 2022. The cohort consisted of 4 males and 28 females with a mean age of 66.47 years (range, 55–88 years). All patients underwent preoperative standing full-length spine x-ray, prone full-length spine CT scout view (FLS-CT), and supine MRI. Among them, 29 patients had single-segment fractures and 3 had double-segment fractures. The local kyphosis Cobb angle (LKCA) was measured on all imaging modalities. The LKCA measured on standing x-ray and FLS-CT were recorded as LKCA_X_ and LKCA_FLSCT_, respectively. On MRI, LKCA was measured on three sagittal slices (left parasagittal, midsagittal, and right parasagittal), recorded as LKCA_LMR_, LKCA_MMR_, and LKCA_RMR_, respectively. Kyphosis flexibility (KF) was calculated based on these measurements. Pairwise comparisons were performed using the Wilcoxon signed-rank test with Bonferroni correction after an overall Friedman test. Equivalence analysis between prone FLS-CT and supine MRI was performed using a prespecified margin of ±5°. Interobserver reliability was assessed using the intraclass correlation coefficient (ICC).

**Results:**

The mean standing LKCA was 39.58 ± 9.00°. The LKCA measured on prone FLS-CT was 29.61 ± 6.96°. On supine MRI, the LKCA values were 28.34 ± 6.37° (LKCA_LMR_), 27.64 ± 6.18° (LKCA_MMR_), and 28.97 ± 5.92° (LKCA_RMR_). The mean LKCA of the three MRI planes was 28.32 ± 5.91°. The corresponding KF values were 24.45% ± 10.86% for prone FLS-CT, 27.36% ± 11.08% for the left parasagittal slice, 29.16% ± 10.89% for the midsagittal slice, 25.52% ± 11.20% for the right parasagittal slice, and 27.35% ± 10.16% for the mean of the three MRI planes. LKCA was significantly lower in the prone and supine positions than in the standing position (all adjusted *p* < 0.001). No significant differences were found between prone FLS-CT and any supine MRI measurement (all adjusted *p* > 0.05). In equivalence analysis, all 95% confidence intervals of the paired mean differences between prone FLS-CT and supine MRI measurements were entirely within the prespecified equivalence margin of ±5°. Interobserver reliability was excellent across all imaging modalities, with ICC values ranging from 0.985 to 0.992.

**Conclusion:**

Kyphosis severity was significantly reduced in the preoperative recumbent position in patients with OTFK. Prone FLS-CT and supine MRI provided clinically comparable estimates of positional kyphosis correction, suggesting that both modalities may be useful for preoperative assessment of kyphosis flexibility in OTFK.

## 1 Introduction

Osteoporotic vertebral fracture is a common condition among the elderly population, leading to the progressive worsening of vertebral wedging deformity. This deformity is mainly caused by the lack of timely intervention and reliance on non-surgical treatments. As a result, kyphosis becomes the ultimate manifestation of this deformity (1). The thoracolumbar vertebral body, where thoracic kyphosis and lumbar lordosis meet, experiences a high level of stress, making it a common location for vertebral fractures (2). When there is secondary kyphosis of the fracture, also known as old thoracolumbar fracture kyphosis (OTFK), and it is accompanied by progressively worsening neurological symptoms and significant sagittal imbalance, corrective osteotomy surgery is often required to restore the optimal curvature of the spine. The current surgical treatment methods depend on measuring spinal pelvic parameters in the preoperative standing position to assess the suitability of osteotomy procedures and identify the specific level at which osteotomy is needed (1, 3). Clinically, posterior column osteotomy (PCO) is commonly utilized in Schwab osteotomy grading for patients with osteotomies for thoracolumbar kyphosis (OTFK). However, three-column osteotomy and osteotomies above it are only suitable for severe cases of kyphosis deformity. The surgical procedure is complex, traumatic, and associated with numerous complications. Therefore, caution should be exercised when using it for patients with OTFK (4–9). Hence, it is crucial to investigate methods that aim to reduce the extent of osteotomy while promoting optimal orthopedic outcomes.

In the context of common spinal deformities, the surgical planning for juvenile idiopathic scoliosis primarily relies on the utilization of preoperative bending images, fulcrum support images, and other assessment techniques to evaluate the flexibility of scoliosis and identify the appropriate fixation segment. Prior research has demonstrated that there are variations in the angles of sagittal and coronal deformities among scoliosis patients in different positions (10–12). However, there is a notable scarcity in examination positions and measurement techniques that adequately assess the flexibility of kyphosis in patients presenting with thoracolumbar fractures and exhibiting favorable spinal flexibility (13, 14). Currently, the assessment of kyphosis severity and kyphotic flexibility is relatively straightforward, wherein spinal parameters are measured using supine MRI and then compared against the corresponding standing spine parameters for evaluation (15, 16).

The full-length spine CT scout view (FLS-CT) position refers to an examination position that has been recently proposed and closely mimics the actual surgical scene. This positioning often results in a certain level of improvement in the spinal deformity and the measured spinal parameters obtained from this position can be utilized for surgical planning. Additionally, it has the potential to reduce the necessity for osteotomy procedures (13). This article aims to compare the kyphosis angles of OTFK patients in different positions, and to provide a reference for the evaluation of spinal flexibility and the planning of surgical plans in OTFK patients in clinical practice.

## 2 Method

### 2.1 Patient sample

A total of 32 patients with osteoporotic thoracolumbar fracture kyphosis were included in this study. All patients underwent standing full-length spine x-ray, supine MRI, and FLS-CT examinations. In this study, the thoracolumbar junction was defined as the region from T11 to L2. The inclusion criteria were as follows: (1) significant loss of vertebral height with a disease course of more than 2 months; (2) significant back pain or lower limb symptoms caused by spinal nerve compression that could not be relieved by conservative treatment; (3) osteoporosis confirmed by dual-energy x-ray absorptiometry or QCT examination; (4) the fractured vertebral body was located between T11 and L2; and (5) complete preoperative imaging data were available. The exclusion criteria were as follows: (1) blurred or invisible vertebral endplates on imaging data that precluded accurate measurement; (2) previous spinal internal fixation surgery; (3) spinal infection or tumor; (4) coronal Cobb angle ≥ 10°; and (5) congenital vertebral deformity or ankylosing spondylitis.

### 2.2 imaging examination method

**Figure 1:**
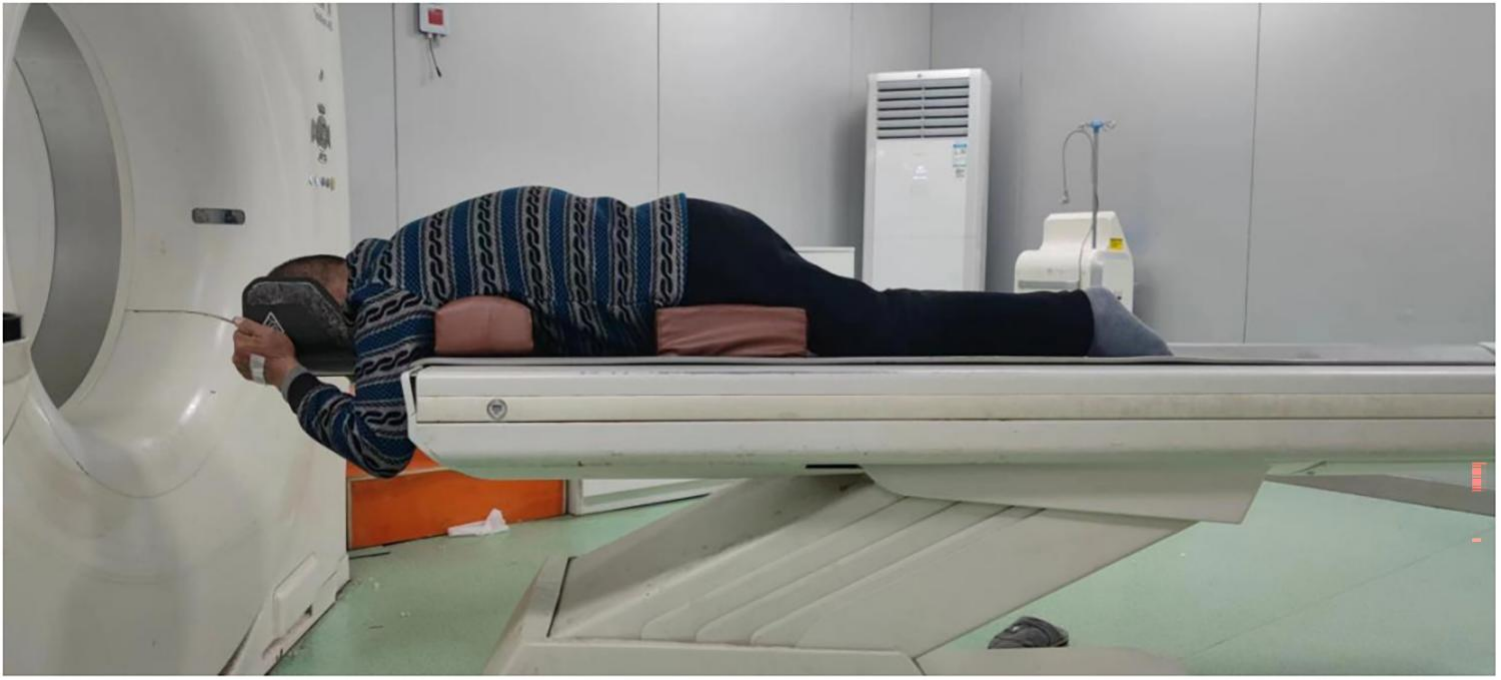
Patient positioning for full-length spine CT scout view.

The specific procedures of the imaging examinations used in this study were as follows. (1) standing full-length spine x-ray: Patients stood erect naturally without support, with arms flexed and fingers touching the clavicles to avoid upper limb overlap with the spinal image. (2) full-length spine CT scout view (FLS-CT): It was taken by NeuViz 128 CT of Neusoft Medical Systems Co., Ltd., and was jointly completed by a spine surgeon and a radiologist. Scanning position: The patient lies prone on the flat panel of the CT instrument, and two surgical position pads with a height of 15 cm are placed on the chest and at the level of the iliac spines on both sides respectively. Both sides after the patient adapts to this position, the lumbar and back muscles will naturally relax without resistance, the chest and abdomen will naturally sag with the help of gravity and pull the thoracolumbar spine forward through the suspension effect. After the degree of back kyphosis spontaneously decreases forward, perform CT scout scan. The scanning range is from the head to the upper 1/3 of the femur, and the scanning parameters are 120 kV, 250 mA. The specific position is shown in Figure 1. (3) MRI in supine position: MRI examinations were performed with the patient in the supine position using a 1.5-T MRI scanner. Sagittal T1-weighted and T2-weighted sequences covering the thoracolumbar region were acquired. Typical imaging parameters were as follows: repetition time (TR) 400–600 ms and echo time (TE) 10–15 ms for T1-weighted images, and TR 3,000–4,000 ms and TE 90–120 ms for T2-weighted images. The slice thickness was 3–4 mm with an interslice gap of 0.5–1 mm.

### 2.3 Imaging and statistical evaluation

Preoperative standing full-length spine radiographs, prone Full-length Spine CT scout view (FLS-CT), and supine MRI were imported into Surgimap software (Nemaris Inc., USA) for measurement of the local kyphosis Cobb angle (LKCA). LKCA was defined as the angle formed between the superior endplate of the cranial vertebra and the inferior endplate of the caudal vertebra spanning the kyphotic segment. The fractured vertebral level was first identified on the standing radiograph, and the same cranial and caudal vertebral endplates were used across all imaging modalities to ensure comparability. In patients with double-level fractures, the kyphotic segment was defined using the most proximal intact superior endplate and the most distal intact inferior endplate encompassing the deformity. For MRI-based assessment, three representative sagittal slices that clearly visualized the vertebral endplates were selected, including the left parasagittal slice, the midsagittal slice, and the right parasagittal slice. These measurements were recorded as LKCALMR, LKCAMMR, and LKCARMR, respectively, and reflected measurement locations rather than differences in patient position. These measurements were recorded as LKCA_LMR_, LKCA_MMR_, and LKCA_RMR_, respectively. The mean value of the three MRI measurements was defined as the MRI mean LKCA.

The standing LKCA, prone FLS-CT LKCA, and MRI LKCA were denoted as LKCA_X_, LKCA_FLSCT_, and LKCA_MRI_, respectively. Kyphosis flexibility (KF) was calculated as KF = (LKCA_X_−LKCA_position_)/LKCAX × 100%, where LKCA_position_ represents the LKCA measured in the corresponding recumbent position. Accordingly, KF values were calculated for prone FLS-CT, the left parasagittal MRI slice, the midsagittal MRI slice, the right parasagittal MRI slice, and the mean of the three MRI planes.

Statistical analysis was performed using SPSS version 26.0 (IBM Corp., USA) and GraphPad Prism version 10.0 (GraphPad Software, USA). Continuous variables are presented as mean ± standard deviation (SD) and 95% confidence intervals (95% CIs). Because LKCA measurements across body positions represented repeated observations within the same patient, overall comparisons were performed using the Friedman test. Pairwise comparisons were performed using the Wilcoxon signed-rank test with Bonferroni correction. To further determine whether prone FLS-CT and supine MRI yielded clinically equivalent LKCA measurements, a paired equivalence analysis was additionally performed with a prespecified equivalence margin of ±5°. Equivalence was concluded when the 95% CI of the paired mean difference was entirely contained within the equivalence bounds. A two-sided *p* value < 0.05 was considered statistically significant.

### 2.4 Interobserver reliability

To assess interobserver reliability, LKCA measurements on standing radiographs, prone FLS-CT, and supine MRI were independently performed by two spine surgeons who were blinded to each other's results. Interobserver agreement was evaluated using the intraclass correlation coefficient (ICC) based on a two-way random-effects model with absolute agreement for single measurements.

## 3 Results

### 3.1 Patient characteristics

A total of 32 patients were included in this study, including 4 males and 28 females. The mean age was 66.47 ± 8.32 years. Among them, 29 patients had single-segment vertebral fractures and 3 patients had double-segment fractures. The baseline characteristics of the patients are summarized in Table 1.

**Table 1:** Patients' demographic and clinical characteristics.

Characteristic	Value
No. of patients	32
Males/Females	4/28
Age (y)	66.47 ± 8.32
Fracture level	
T11	3
T12	15
T12 and L1	3
L1	9
L2	2

### 3.2 LKCA and kyphosis flexibility across body positions

The preoperative standing LKCA was 39.58 ± 9.00°. The prone FLS-CT LKCA was 29.61 ± 6.96°. On supine MRI, the LKCA values were 28.34 ± 6.37° for the left parasagittal slice, 27.64 ± 6.18° for the midsagittal slice, and 28.97 ± 5.92° for the right parasagittal slice. The mean LKCA of the three MRI planes was 28.32 ± 5.91°. The corresponding KF values were 24.45% ± 10.86% for prone FLS-CT, 27.36% ± 11.08% for the left parasagittal slice, 29.16% ± 10.89% for the midsagittal slice, 25.52% ± 11.20% for the right parasagittal slice, and 27.35% ± 10.16% for the mean of the three MRI planes. Detailed LKCA and KF results, together with 95% CIs, are summarized in Table 2.

**Table 2:** Local kyphosis cobb angle (LKCA) and kyphosis flexibility (KF) measured in different body positions.

Position	LKCA (°) mean ± SD	LKCA 95% CI (°)	KF (%) mean ± SD	KF 95% CI (%)
Standing x-ray	39.58 ± 9.00	36.34–42.83	-	-
Prone FLS-CT	29.61 ± 6.96*	27.10–32.12	24.45 ± 10.86	20.54–28.37
Supine MRI-L	28.34 ± 6.37*	26.05–30.64	27.36 ± 11.08	23.37–31.36
Supine MRI-M	27.64 ± 6.18*	25.41–29.87	29.16 ± 10.89	25.24–33.09
Supine MRI-R	28.97 ± 5.92*	26.83–31.10	25.52 ± 11.20	21.48–29.56
Supine MRI mean	28.32 ± 5.91*	26.19–30.45	27.35 ± 10.16	23.68–31.01

LKCA, local kyphosis Cobb angle; KF, kyphosis flexibility, calculated as (LKCA_X_−LKCA_supine/prone_)/LKCA_X_ × 100%.

The symbol “*” indicates a statistically significant difference compared with Standing x-ray (*p* < 0.05).

### 3.3 Between-position comparisons and equivalence analysis

Overall comparison of LKCA across body positions showed a significant difference by Friedman test. Pairwise comparisons using the Wilcoxon signed-rank test with Bonferroni correction demonstrated that the standing LKCA was significantly greater than the LKCA measured on prone FLS-CT and on all supine MRI planes (all adjusted *p* < 0.001). In contrast, no significant differences were found between prone FLS-CT and any of the supine MRI measurements, and no significant differences were found among the three sagittal MRI measurements themselves (all adjusted *p* > 0.05) (Table 3).

**Table 3:** Pairwise comparisons of LKCA among different body positions.

Position	Standing x-ray	Prone FLS-CT	Supine MRI-L	Supine MRI-M	Supine MRI-R	Supine MRI mean
Standing x-ray	-	<0.001*	<0.001*	<0.001*	<0.001*	<0.001*
Prone FLS-CT	<0.001*	-	1	1	1	1
Supine MRI-L	<0.001*	1	-	1	0.942	1
Supine MRI-M	<0.001*	1	1	-	0.743	1
Supine MRI-R	<0.001*	1	0.942	0.743	-	0.781
Supine MRI mean	<0.001*	1	1	1	0.781	-

Values are Bonferroni-adjusted *p* values from Wilcoxon signed-rank tests following an overall Friedman test.

* *P* < 0.05.

**Figure 2:**
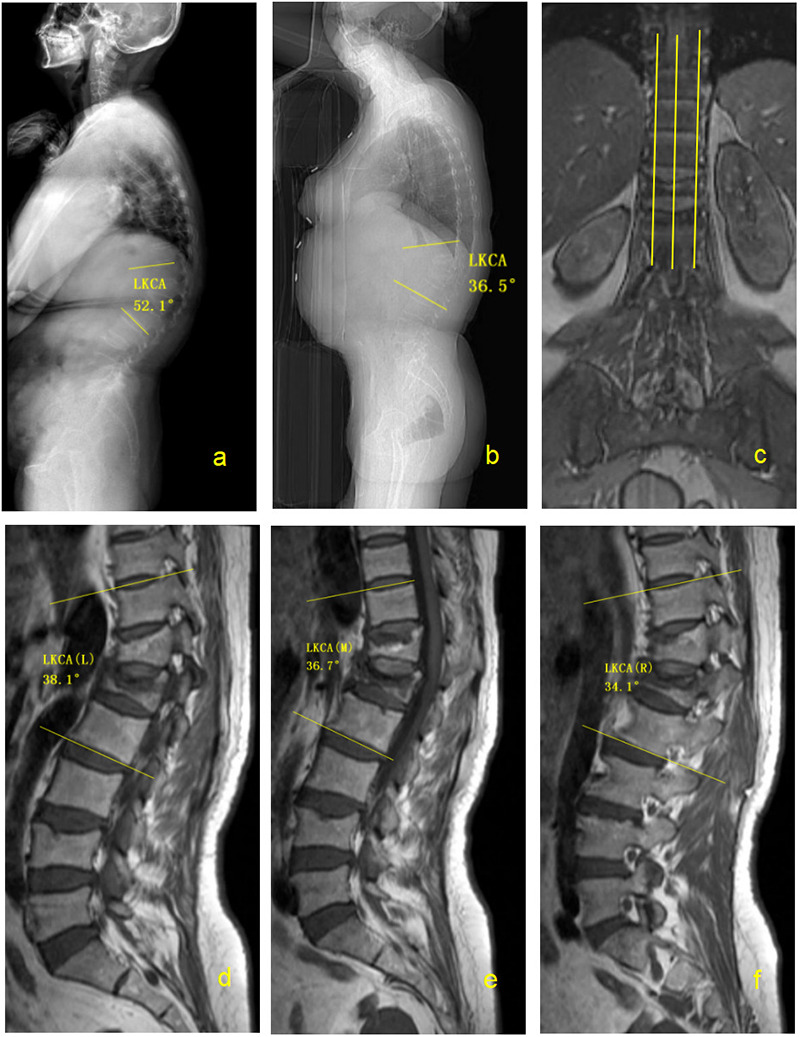
Representative case of a 68-year-old female patient with kyphosis secondary to osteoporotic thoracolumbar fracture kyphosis (OTFK). **(a)** Preoperative standing radiograph showing a standing LKCA of 52.1°. **(b)** Full-length spine CT scout view (FLS-CT) obtained in the prone position showing an LKCA of 36.5°. **(c)** Schematic illustration of the three sagittal MRI slices used for LKCA measurement. **(d)** MRI left parasagittal slice showing an LKCA of 38.1°. **(e)** MRI midsagittal slice showing an LKCA of 38.7°. **(f)** MRI right parasagittal slice showing an LKCA of 34.1°.

For equivalence analysis, the mean paired differences between prone FLS-CT and supine MRI-L, MRI-M, MRI-R, and the mean of the three MRI planes were 1.27°, 1.97°, 0.64°, and 1.29°, respectively. All corresponding 95% CIs were entirely contained within the prespecified equivalence margin of ±5°, supporting clinical equivalence between prone FLS-CT and supine MRI measurements in this cohort (Table 4). A representative case is shown in Figure 2.

**Table 4:** Equivalence analysis between prone FLS-CT and supine MRI measurements.

Comparison	Mean paired difference (°)	95% CI (°)	Equivalence margin (°)	Equivalent
Prone FLS-CT vs. Supine MRI-L	1.27	−0.43–2.97	±5	Yes
Prone FLS-CT vs. Supine MRI-M	1.97	0.16–3.77	±5	Yes
Prone FLS-CT vs. Supine MRI-R	0.64	−1.08–2.36	±5	Yes
Prone FLS-CT vs. Supine MRI mean	1.29	−0.34–2.92	±5	Yes

Mean paired difference was calculated as prone FLS-CT minus the corresponding supine MRI measurement. Equivalence margin was preset at ±5°. Equivalence was concluded when the 95% CI was entirely within −5° to +5°.

### 3.4 Interobserver reliability of LKCA measurements

Interobserver reliability analysis showed excellent agreement for LKCA measurements across all evaluated imaging modalities. The ICC was 0.992 (95% CI, 0.987–0.995) for standing radiographs and 0.990 (95% CI, 0.983–0.994) for prone FLS-CT. On supine MRI, the ICC values were 0.985 (95% CI, 0.974–0.991) for the left parasagittal slice, 0.989 (95% CI, 0.980–0.992) for the midsagittal slice, and 0.985 (95% CI, 0.974–0.990) for the right parasagittal slice, indicating excellent measurement reproducibility between the two observers (Table 5).

**Table 5:** Interobserver reliability of LKCA measurements across imaging modalities.

Measurement	ICC	95% CI
Standing x-ray	0.992	0.987–0.995
Prone FLS-CT	0.990	0.983–0.994
Supine MRI-L	0.985	0.974–0.991
Supine MRI-M	0.989	0.980–0.992
Supine MRI-R	0.985	0.974–0.990
Supine MRI mean	0.993	0.987–0.996

ICC was calculated using a two-way random-effects model with absolute agreement for single measurements.

## 4 Discussion

Old thoracolumbar fracture kyphosis (OTFK) is predominantly characterized by angular kyphosis with a substantial kyphosis angle. For patients presenting severe sagittal imbalance, surgical intervention commonly entails posterior spinal osteotomy orthopedic fixation (17, 18). The primary objective of the surgical procedure is to reinstill three-dimensional spinal stability and alleviate manifestations of lumbar pain, concurrently rectifying LKCA and reestablishing sagittal balance in the spinal column (15, 19, 20). Currently, the posterior column osteotomy in the Schwab osteotomy classification is the favored surgical option, albeit with limited orthopedic efficacy (21). However, it is worth noting that patients with OTFK mostly comprise elderly individuals burdened with multiple complications and exhibiting poor surgical tolerance. Consequently, the undertaking of three or more column osteotomies in such patients entails a high risk (5, 22). Hence, when devising the preoperative surgical strategy for OTFK patients, it is imperative to prioritize both orthopedic efficacy and minimize surgical trauma. Therefore, when planning surgery for OTFK, the extent of osteotomy should be minimized whenever adequate correction can still be achieved.

Previous research has indicated that alterations in the positioning of scoliosis patients during surgery have an impact on the scoliosis curve, with over half of the overall correction attained during surgery being attributed to changes in position and anesthesia (23, 24). The surgical correction protocol for scoliosis is based on the utilization of preoperative standing x-rays. However, conventional posterior surgery patients typically assume a prone position rather than standing or supine positions. Additionally, the implementation of muscle relaxants, muscle incision, and soft tissue release techniques can alleviate scoliosis to varying extents. As a result, the intraoperative lordosis and scoliosis may differ from the preoperative condition, thereby leading to imprecise surgical planning (10, 15, 22). When the angle of spinal deformity decreases during the operation, the level of surgical osteotomy and the necessity of osteotomy should also be reconsidered. Because spinal flexibility is generally better in AIS, a variety of preoperative assessment methods are used to estimate the extent of spontaneous correction and to reduce the number of fixation levels when appropriate (25, 26). In contrast, there are fewer established methods for assessing spinal flexibility in patients with OTFK.

Kaiser et al. (27) employed preoperative MRI and standing lateral x-ray measurements of thoracic kyphosis (TK) to evaluate the spinal flexibility in cases of Scheuermann's kyphosis. Their findings revealed that for flexible curves, the posterior approach is typically adequate for correcting the spinal deformity. However, for rigid spinal curves, anterior release and posterior fusion are often necessary, and in some cases, anteroposterior multi-segment Ponte osteotomy may be required. Karikari et al. (28) employed the preoperative supine x-ray to assess spinal flexibility by measuring the LL angle. The patients were categorized into two groups, namely rigidity and flexibility, and further subdivided into either a three-column osteotomy group or a non-three-column osteotomy group. It was observed that even among patients in the flexible group who did not undergo three-column osteotomy, the postoperative sagittal plane deformity was still ameliorated. This implies that adjusting the level of osteotomy does not impact the clinical efficacy or postoperative imaging parameters. Lovecchio et al. (29) used the supine vs. standing PI-LL recovery ratio to assess spinal flexibility and found that patients with higher flexibility improved lumbar lordosis with intraoperative positioning alone, eliminating the need for larger corrective surgery. Therefore, the flexibility of the kyphotic curve can determine the choice of whether to perform osteotomy and the level of osteotomy, but there is no uniform standard for the measurement and evaluation of spinal flexibility (29, 30).

In this study, LKCA was measured in the standing, prone, and supine positions, and substantial positional correction of kyphosis was observed in both recumbent positions. Compared with the standing radiograph, the mean LKCA decreased by 9.97° in the prone position and by 11.24°, 11.94°, and 10.61° on the left parasagittal, midsagittal, and right parasagittal MRI slices, respectively. The corresponding KF values also indicated appreciable positional flexibility. These findings suggest that part of the kyphotic deformity in OTFK is position-dependent rather than completely fixed, which is clinically relevant when estimating the extent of correction that may be achieved without more aggressive osteotomy. Importantly, the present study did not only show that there was no statistically significant difference between prone FLS-CT and supine MRI measurements; the equivalence analysis further demonstrated that the paired differences between prone FLS-CT and all supine MRI measurements were small, and all 95% confidence intervals were entirely contained within the prespecified equivalence margin of ±5°. Therefore, prone FLS-CT and supine MRI may be regarded as clinically comparable tools for evaluating kyphosis flexibility in this cohort. In addition, although slight variation was observed among different sagittal MRI slices, no significant difference was found among the three MRI planes, suggesting that the overall assessment of positional kyphosis correction on MRI remained stable across slices.

At present, the two examination methods used to assess spinal flexibility in this study each have practical advantages and limitations. Supine MRI is routinely performed before spinal surgery, allows the paraspinal musculature to relax in the recumbent position, and can reflect positional reduction of kyphosis without additional radiation exposure. In addition, MRI provides information on neural compression, vertebral morphology, and soft tissue structures, which is valuable for comprehensive preoperative evaluation. However, in OTFK, vertebral height loss and endplate irregularity may lead to slight differences in LKCA measurements across sagittal slices, which explains why three representative MRI planes were assessed in the present study. In addition, to ensure clear visualization of the complete vertebral endplates required for measurement on MRI, no patients with substantial scoliosis were included in this study. The use of CT scout images for sagittal alignment assessment was first introduced by Weisz (31) in 2014 in patients with flat back deformity. Compared with conventional x-ray examination, the radiation exposure associated with CT scout imaging is substantially lower. In the present study, the radiation dose of the CT scout image was lower than that of standard radiography and was approximately one-sixth under our institutional protocol. When soft pads are placed under the chest and pelvis, the patient is positioned prone, allowing the abdomen to hang freely and the thoracolumbar spine to extend anteriorly, thereby permitting assessment of positional kyphosis correction. The measured spinal parameters may therefore provide useful information for surgical planning (30). However, because image quality may be affected by soft-tissue overlap and scout-view artifacts, some cases with poor image quality had to be excluded (32). Spinal hyperextension radiographs are commonly used to assess intervertebral stability, and although segmental angulation (SA) has been well studied, the subvertebral kyphosis angle has been less frequently evaluated (33–35). In the present study, hyperextension radiographs were not included because patients with OTFK often have severe low back pain, and this examination may increase discomfort, reduce patient cooperation, and compromise measurement accuracy. In addition, because not all patients underwent surgery, spinal alignment under preoperative anesthesia was not evaluated in the present study.

Another important finding of the present study was the high interobserver reproducibility of LKCA measurements across all imaging modalities. The ICC values for standing radiographs, prone FLS-CT, and supine MRI were all within the excellent range, indicating that the measurement protocol based on predefined cranial and caudal endplates was highly consistent between observers. This strengthens the robustness of the present results and supports the reliability of LKCA as the primary radiographic parameter for comparing positional kyphosis correction in OTFK.

This study has several limitations. First, this was a retrospective single-center study with a relatively small sample size, which limits the generalizability of the findings. Second, although equivalence analysis was performed using a prespecified margin of ±5°, the sample size was not determined prospectively for equivalence testing. Third, the present analysis focused on preoperative positional imaging and did not directly correlate prone FLS-CT correction with intraoperative or postoperative correction outcomes. In addition, only patients without substantial coronal deformity were included, which may limit the applicability of these findings to more complex deformity patterns. Despite these limitations, the present study suggests that prone FLS-CT and supine MRI provide clinically comparable estimates of positional kyphosis correction in patients with OTFK. These findings may be useful for preoperative assessment of kyphosis flexibility, although further validation in larger prospective studies is still required.

## 5 Conclusions

In patients with osteoporotic thoracolumbar fracture kyphosis, prone FLS-CT and supine MRI provided clinically comparable estimates of positional kyphosis correction. Both modalities may be useful for preoperative assessment of kyphosis flexibility and may help refine osteotomy planning.

## Data Availability

The raw data supporting the conclusions of this article will be made available by the authors, without undue reservation.
